# CRISPR/Cas9 Mediated Deletion of the Uox Gene Generates a Mouse Model of Hyperuricemia with Multiple Complications

**DOI:** 10.1007/s12265-024-10526-6

**Published:** 2024-06-10

**Authors:** Linzi Zeng, Shalaimaiti Shali, Yabiao Gao, Xingchen Du, Xiaoxia Zhu, Lin Li, Yuxiang Dai, Ping Zhou

**Affiliations:** 1 Department of Physiology and Pathophysiology of School of Basic Medical Sciences, Department of Cardiology, Zhongshan Hospital, Fudan University, Shanghai, China; 2 Shanghai Institute of Cardiovascular Diseases, Shanghai, China; 3 National Clinical Research Center for Interventional Medicine, Shanghai, China; 4 Division of Rheumatology, Huashan Hospital, Fudan University, Shanghai, China; 5 Institute of Rheumatology, Immunology and Allergy, Fudan University, Shanghai, China; 6 Department of Nephrology, Shanghai Changzheng Hospital, The Second Affiliated Hospital of Naval Medical University, Shanghai, China

**Keywords:** Urate oxidase, CRISPR/Cas9, Hyperuricemia, Mouse model, C57BL/6J

## Abstract

Hyperuricemia is a common metabolic disorder with severe complications. We aimed to develop a mouse model for spontaneous hyperuricemia. *Uox*^-/-^ mouse model was generated on C57BL/6J background by deleting exon 2-4 of *Uox* using the CRISPR/Cas9 system. The prototypic *Uox* ^-/-^mice had 5.5-fold increased serum uric acid (1351.04±276.58μmol/L) as compared to the wild type mice (*P*<0.0001), but died by 4 weeks. After allopurinol (3ug/g) intervention, they all survived > 8 weeks. The serum uric acid was 612.55±146.98μmol/L in the 8-week-old allopurinol-rescued *Uox* ^-/-^mice, which manifested multiple complications including severe renal insufficiency, hypertension, left ventricular remodeling and systolic dysfunction, aortic endothelial dysfunction, hepatic steatosis and elevated liver enzymes, as well as hyperglycemia and hypercholesteremia. The present *Uox*^*-/-*^ mice developed spontaneous hyperuricemia complicated with urate nephropathy, cardiovascular disease and cardiometabolic disorders, and may provide a novel tool to study hyperuricemia associated early-onset cardiovascular disorders in human.

**Graphical Abstract:**

A mouse model of hyperuricemia with multiple complications constructed by knocking out of urate oxidase (Uox) using CRISPR/Cas9 technology. Uox-/-: homozygous; Uox+/-: heterozygous; SUA: serum uric acid; ALT: alanine aminotransferase; AST: aspartate aminotransferase.

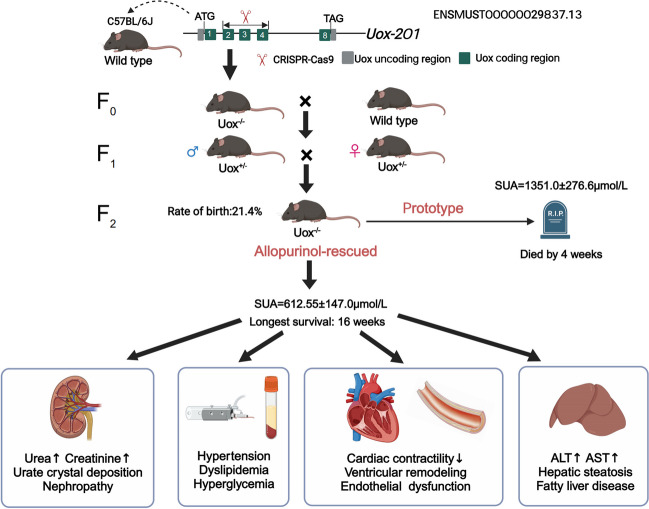

**Supplementary Information:**

The online version contains supplementary material available at 10.1007/s12265-024-10526-6.

## Introduction

Hyperuricemia, characterized by increased concentration of serum uric acid (SUA) above 420 μmol/L, has been an increasingly common metabolic disorder with a precipitous increase particularly among young adults [1, 2]. It is the major causal risk factor of gout and urate-nephropathy, and often entangles with a cluster of cardiometabolic disorders including hypertension, diabetes mellites, dyslipidemia and nonalcoholic fatty liver disease (NAFLD), which could jointly contribute to the development of cardiovascular disease [3]. However, the independent causal effect of hyperuricemia on these metabolic comorbidities and cardiovascular disease remains largely obscure [4]. Therefore, murine models to replicate early-onset hyperuricemia in humans is of paramount importance to better understand the progression trajectories and underlying mechanisms, and to develop effective medications.

Uric acid (UA) is an end product of purine metabolism that is specific to humans due to silencing of the gene encoding urate oxidase (Uox) or uricase during primate evolution. Unlike human, most mammals have active Uox that further degrades UA to allantoin, and therefore challenges exist in the establishment of efficient and sustainable animal model of hyperuricemia using pharmaceutical or dietary-induced approaches [5]. Given the major difference in Uox gene expression between human and mice, genetic modifications that target *Uox* either by homologous recombination in embryonic stem cells [6] or by using transcription activator-like effector nuclease mediated deletion have been used to generate Uox knockout (*Uox*^-/-^) mouse model with spontaneously high SUA concentrations [7]. However, both embryonic lethality and postnatal mortality are the major hurdles of these *Uox* deficient mice. Alternatively, CRISPR/Cas9 is an advanced technology that facilitates more precise genetic engineering. The uricase-deficient “Kunming-DY rats” with stable hyperuricemia and better survival is an example of such an approach [8]. Herein, we established a *Uox*^-/-^ mouse model on C57BL/6J genetic background by deleting the exon 2-4 of *Uox* using CRISPR/Cas9 system. In this study, we present the hyperuricemic phenotypes regarding magnitude of serum UA elevation, urate nephropathy, cardiometabolic abnormalities, as well as cardiovascular and hepatic complications.

## Materials and Methods

### Construction of the *Uox*^-/-^ Mouse Model

C57BL/6J Mice were raised in the Department of Laboratory Animal Science of Shanghai Medical college of Fudan University in a specific pathogen free environment at 22^°^C, with a humidity of 45%-55%, under 12-hour light-dark cycle, and with free approach to food and water.

The Uox gene is located on chromosome three, and had eight exons and six transcripts. Two gRNA fragments (gRNA1: 5’-GTTAACTCCAAACTATATAG-3’; gRNA2: 5’-GGTTACTGGATCATTGGTAC-3’) targeting at three exons (exon 2-4) of the Uox-201 transcript were generated by in vitro transcription, and then co-injected together with commercially available Cas9 protein into fertilized C57BL/6J mouse eggs. Subsequently, these eggs were transplanted into the oviducts of pseudopregnant female mice for embryonic development. *Uox*^-/-^ mice (F_0_) were matched with Wild type (WT) mice to generate heterozygous *Uox*^*+*/-^ mice (F_1_). Finally, homozygous *Uox*^-/-^ (F_2_) offspring were generated by heterozygous mating (Graphical abstract).

**Fig. 1:**
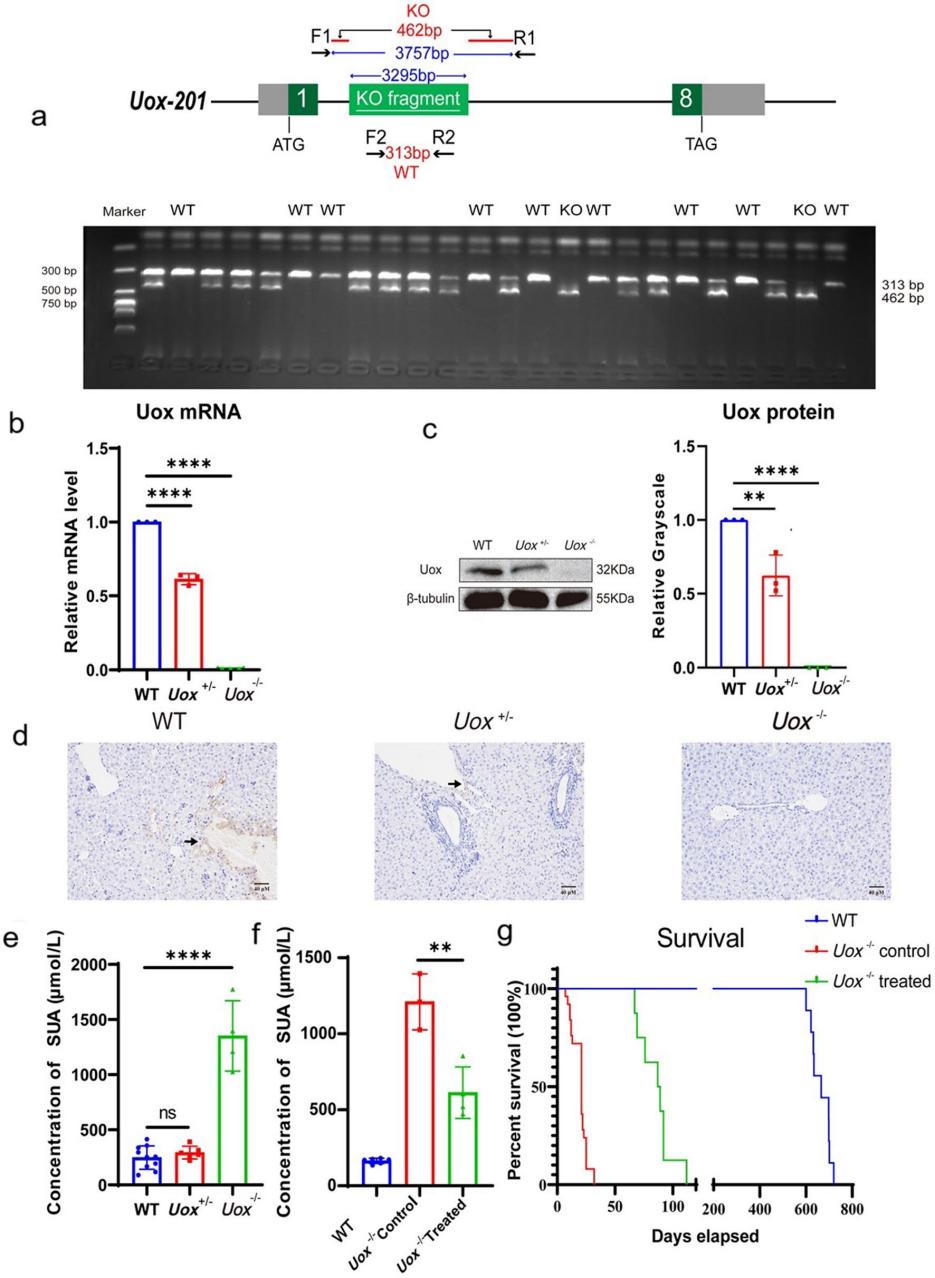
The construction *Uox*^-/-^ mice with spontaneous hyperuricemia a. Genotyping of uric oxidase (Uox) knockout (KO) and wild type (WT) mice by DNA gel electrophoresis. b. Hepatic expression of Uox mRNA determined by quantitative reverse-transcription polymerase chain reaction. c. Hepatic expression of Uox protein determined by Western blot. d. Hepatic expression of Uox determined by immunohistochemistry using anti-Uox antibodies, scale Bar=40μm. e. The concentration of serum uric acid at three weeks of age (*n*=4 (two female, two male)). f. Allopurinol treatment reduces serum uric acid in Uox^-/-^ mice at eight weeks of age (*n*=4 (three female, one male)). g. Allopurinol treatment prolongs survival of the *Uox*^-/-^ mice (*n*=8, five female, three male). *Uox*^-/-^: homozygous; *Uox*^+/-^: heterozygous. Data were expressed as mean ±SEM. **: *P* < 0.05, ****: *P* < 0.0001

Genotyping was conducted at one week after birth by polymerase chain reaction (PCR) amplification of complementary DNA (cDNA) obtained from tail tissue (Supplementary materials). The PCR products were separated by DNA electrophoresis at 313 base pairs (bp) for WT allele, at 462bp for homogeneous *Uox*^-/-^ allele, while at both 313bp and 462bp for heterogeneous *Uox*^+/-^ allele respectively, suggesting 3,295 bp would be deleted. (Fig. 1a).

### Quantitative RT-PCR and Western Blotting

Total RNA was isolated from the liver tissue using Trizol reagent (Vazyme, Nanjing, China) and then reverse-transcribed into cDNA using a Hiscript III Reverse Transcriptase Kit (Vazyme, Nanjing, China). The sequencings of primers were 5’-GGACCTGACTGACTACCTCAT-3’ (β-actin-forward), 5’-GGACCTGACTGACTACCTCAT-3’ (β-actin-reverse), 5’-CCCAGGCTAAACTCTCAGGCT-3’ (Uox-forward), and 5’-TGTCAGGGAAACAGTCATTTCACA-3’ (Uox-reverse). Real-time quantitative PCR was performed using AceQ qPCR SYBR Green Master Mix (Vazyme, Nanjing, China) on a Fluorescent Quantitative PCR (Biored, California, USA) with the following parameters: 94°C five minutes, 94^°^C30 seconds, 60°C 30 seconds, 72^°^C one minute, 35 cycles. The threshold cycle (Ct) was determined and used to calculate ΔCT values. The ΔΔCt (2-ΔΔCt) was used to calculate relative mRNA expression, with each measurement performed in triplicate.

Uox protein expression was determined by Western blot using anti-Uox primary antibody (at 1:500; Santa Cruz Biotechnology, Dallas, TX) and anti-β-tubulin antibody (at 1:10000; ptoteintech, Wuhan, China). The membranes were then incubated with horseradish peroxidase-conjugated anti-mouse IgG (1:10000; CST, Boston, USA). Each measurement was performed in triplicate, and all results were normalized against β-tubulin.

### Serum Biochemical Analysis

Blood sampling was performed from the outer canthus of anesthetized mice after overnight fasting. After one hour incubation at a room temperature, serum was obtained by spinning at 3,000×g and at 4^°^C for five minutes. SUA was measured using uric acid assay kit (Abcam, Cambridge, UK) following the protocol. Other serum biochemical indicators including, urea, creatinine, aspartate aminotransferase (AST), alanine aminotransferase (ALT), total cholesterol (TC), triglyceride (TG), low-density lipoprotein cholesterol (LDL-C), high density lipoprotein cholesterol (HDL-C) and fasting glucose were determined using an automatic biochemical analyzer (Hitachi, Tokyo, Japan).

### Blood Pressure and Echocardiographic Measurement

The blood pressure (BP) measurements were conducted using the BP-2000 non-invasive BP analysis system (Visitech system, Drammen, Norway) with methods adhering to the provided instructions. Echocardiographic examination was performed using two-dimensional and M-mode imaging by the high-resolution real-time ultrasound pre-clinical imaging system (Fujifilm Visual Sonics, Toronto, Canada) in the parasternal long-axis view. Dimensional and functional parameters of the left ventricle were measured at the level of the papillary muscles.

### Histological Analysis

Tissue sections of the kidney, heart, aorta and liver were subjected to hematoxylin-eosin (HE) staining, and visualized under fluorescence microscope in a light mode. Kidney sections were also stained by Masson’s trichrome. Besides, Wheat Germ Agglutinin (WGA) staining, Van Gieson (VG) staining and Oil Red O Staining were performed on the tissues from heart, aorta and liver, respectively. Moreover, immunohistochemical analyses were conducted using primary antibodies as follows: Uox (1:100; Santa Cruz Biotechnology, Dallas, TX), lymphocyte CD3 (1:1000, Proteintech, Wuhan, China), macrophage CD68 (1:1000, Proteintech, Wuhan, China), proliferating cell nuclear antigen (PCNA) (1;1000, Proteintech, Wuhan, China), ZO1 (1:300, Abmart, Shanghai, China) and Ve-cadherin (1:200, R&D Systems, Minneapolis, MN, USA).

### Statistical Analysis

All quantitative values were presented in the form of mean ± standard error of mean (SEM) or median with the interquartile ranges (IQR) as appropriate, and the differences between groups were analyzed by either Student’s t-test or Mann–Whitney non-parametric tests. Categorical variables were presented as absolute values (percentages). Survival analysis was analyzed by Log-rank test. *P* < 0.05 was considered statistically significant.

## Results

### Generation of *Uox*^-/-^ Mice with Spontaneous Hyperuricemia

Both mRNA and protein expressions of Uox were absent in the liver of *Uox*^-/-^ mice , but not in the *Uox*^+/-^ and WT mice (Fig. 1b-d). Notably, *Uox*^-/-^ mice (3 weeks old) had an extremely high SUA concentration (1351.04±276.58μmol/L), which was 5.5-fold higher than that in WT mice (248.19±100.59μmol/L, *P*<0.0001) (Fig. 1e). Albeit significant reduction of *Uox* hepatic expression (*P*=0.0091, Fig. 1b-d), the *Uox*^+/-^ mice (292.60±52.42μmol/L) had unchanged levels of SUA as compared to WT mice (*P*=0.364, Fig. 1e).

The birth rate of *Uox*^-/-^ mice was at 21.35% (114 of 534), slightly lower than the expected Mendelian frequency, suggesting either embryonic lethality or neonatal death in the first week. Besides, they barely survived to four weeks (median: 3.0 weeks, IQR:1.7 to 3.6 weeks). However, daily gastric administration of allopurinol (3μg/g) [6] after genotyping not only reduced the concentrations of SUA by 54.75% (612.55±146.98μmol/L) to the similar levels in human hyperuricemia (*P*=0.0067, allopurinol-treated vs. not treated, Fig. 1f), but also improved their survivals up to 16 weeks (median: 12.7 weeks, IQR: 9.9-13.4 weeks, *P*<0.0001, Fig. 1g). Hence, the following phenotypic examinations were performed on the allopurinol-treated *Uox*^-/-^ mice at the age of eight weeks.

### Renal Dysfunction and Nephropathy

**Fig. 2:**
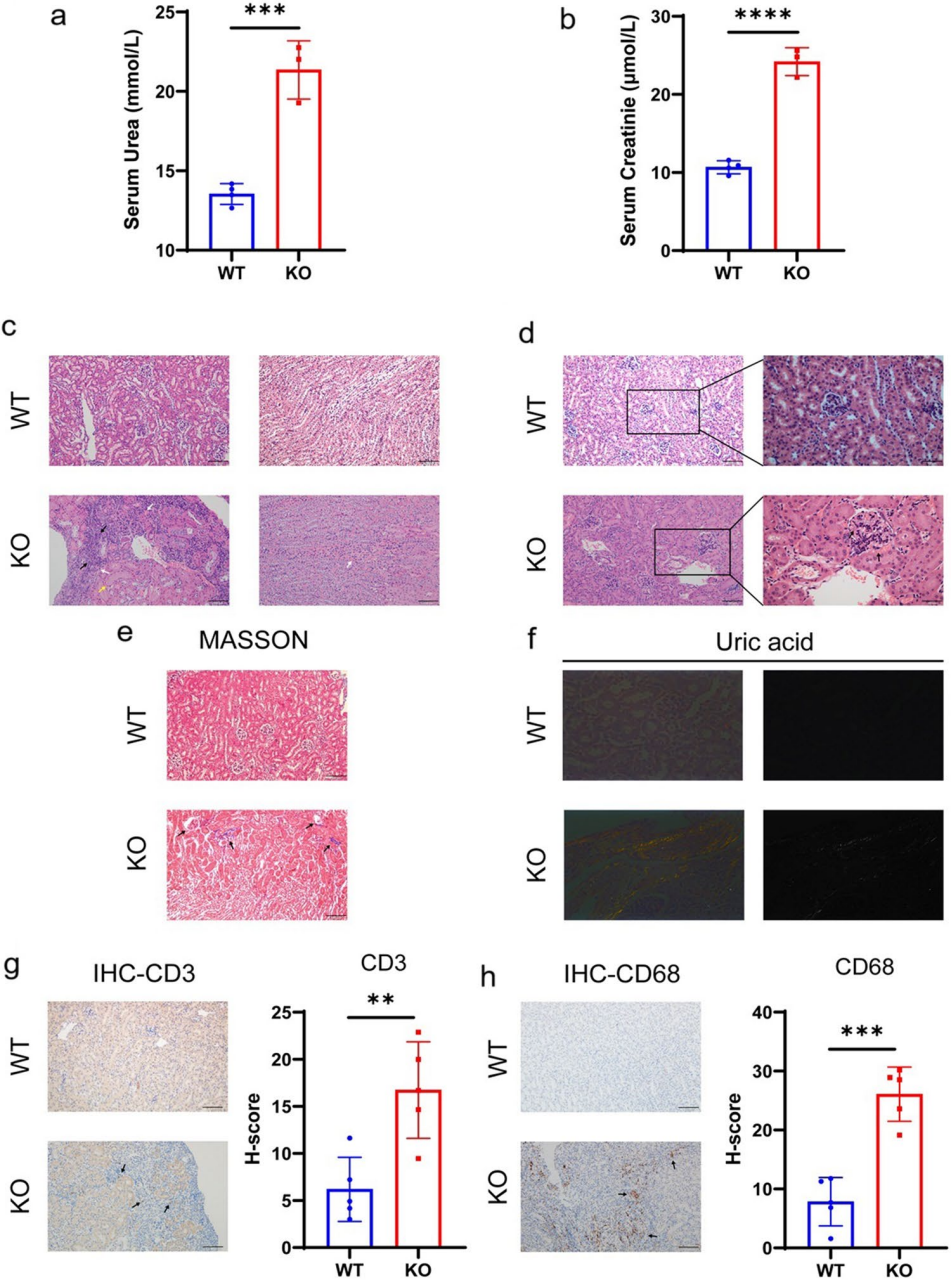
Renal dysfunction and urate-nephropathy in the allopurinol-rescued *Uox*^-/-^ mice at eight weeks after birth a. Serum concentration of urea (*n*=3 (female)); b. Serum concentration of creatinine (*n*=3 (female)); c. Histological examination of the kidney by HE staining; black arrow: massive interstitial infiltration of mononuclear cells; white arrow: interstitial and medullar hemorrhage; yellow arrow: degeneration and necrosis of tubular epithelial cells, tubular dilation with tubular amyloidosis and casts; d. Representative images of HE staining; black arrow: glomeruli enlargement with thronged erythrocytes and decreased capsular space; e. Representative images of renal Masson’s trichrome staining; black arrow: interstitial fibrosis; f. Presence of urate crystal deposition under polarized light; g. Lymphocyte infiltration as determined by immunohistochemistry with anti-CD3 antibodies (*n*=5 (four female, one male)); h. Macrophage infiltration as determined by immunohistochemistry with anti-CD68 antibodies (*n*=5 (four female, one male)). Scale bar = 100μm; data were expressed as mean ± SEM, **: *P* < 0.01, ***: *P* < 0.001, ****: *P* < 0.0001

The glomerular filtration function was compromised in the *Uox*^-/-^ mice, as indicated by elevated levels of blood urea (21.35±1.50 *vs.* 13.54±0.57 mmol/L, *P*<0.0005) and serum creatinine (24.20±1.45 *vs.* 10.68±0.72mmol/L, *P*<0.0001) compared to WT controls (Fig. 2a-b). Histological examination revealed severe kidney structural abnormalities. To begin, the tubular walls were thin, showing necrotic epithelial cells with evident karyopyknosis; the tubules were also dilated and filled with massive granular casts along with amyloid exudation; besides, severe interstitial bleeding in the renal medulla was also common (Fig. 2c). In addition, there was significant glomeruli enlargement with thronged erythrocytes and decreased capsular space; the mesangial expansion and increased eosinophilic matrix were also frequently detected (Fig. 2d). Moreover, the significant tubulointerstitial fibrosis and glomerulosclerosis were commonly observed (Fig. 2e), and so were the urate crystal deposits under polarized light microscope (Fig. 2f). Importantly, there were small amount of (CD3+) T lymphocytes (*P*=0.0050), and massive (CD68+) macrophages (*P*=0.0002) infiltration, indicating substantial corticomedullar inflammation (Fig. 2g-h).

### Hypertension and Cardiometabolic Disturbance

**Fig. 3:**
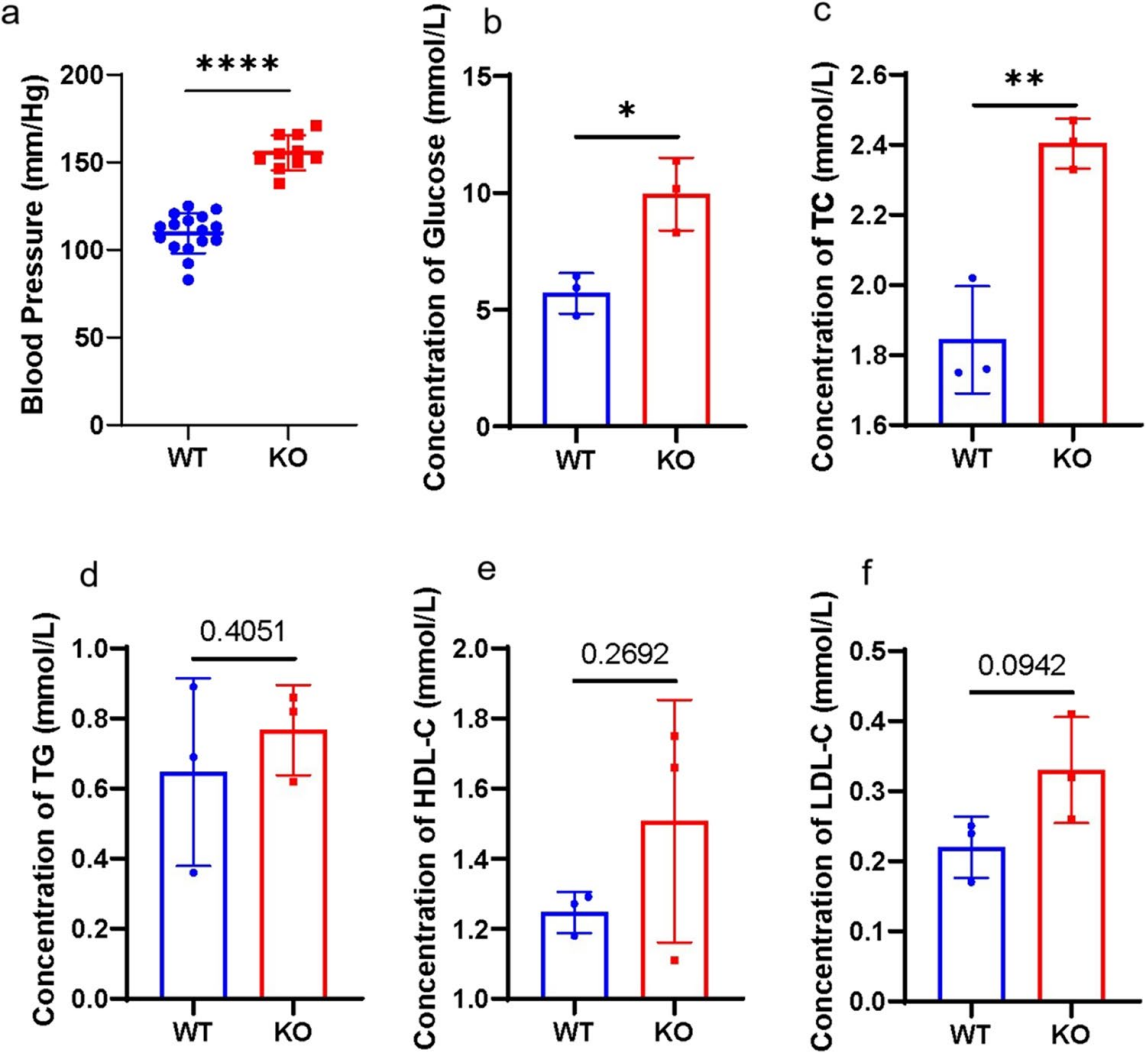
Hypertension and cardiometabolic abnormalities in the allopurinol-rescued *Uox*^-/-^ mice at eight weeks after birth a. The systolic blood pressure (*n*=10, eight female, two male); b. Serum concentration of fasting glucose (*n*=3 (female)); c. Serum concentration of total cholesterol (TC) (*n*=3 (female)); d. Serum concentration of fasting triglycerides (TG) (*n*=3 (female)); e. Serum concentration of high-density lipoprotein cholesterol (HDL-C) (*n*=3 (female)); f. Serum concentration of high-density lipoprotein cholesterol (LDL-C) (*n*=3 (female)). Data were expressed as mean ±SEM. *: *P* < 0.05, **: *P* < 0.01, ****: *P* < 0.0001

The systolic BP was significantly higher in the *Uox*^-/-^ mice (155.25±9.48mmHg) than in the WT controls (109.53±11.02mmHg, *P*< 0.0001), indicating that the *Uox*^-/-^ mice developed early-onset hypertension (Fig. 3a). Also, biochemical analysis confirmed the deleterious effects of hyperuricemia on both glucose and lipid profiles. Compared with WT counterparts, the *Uox*^-/-^ mice had significantly elevated fasting glucose (*P*=0.0146) and TC (*P*=0.0045) (Fig. 3b-c); besides, there was a trend towards elevated levels of TG and LDL-C, and decreased levels of HDL-C in the *Uox*^-/-^ mice, although not statistically significant (all *P*>0.05, Fig. 3d-f).

### Cardiovascular Complications

**Fig. 4:**
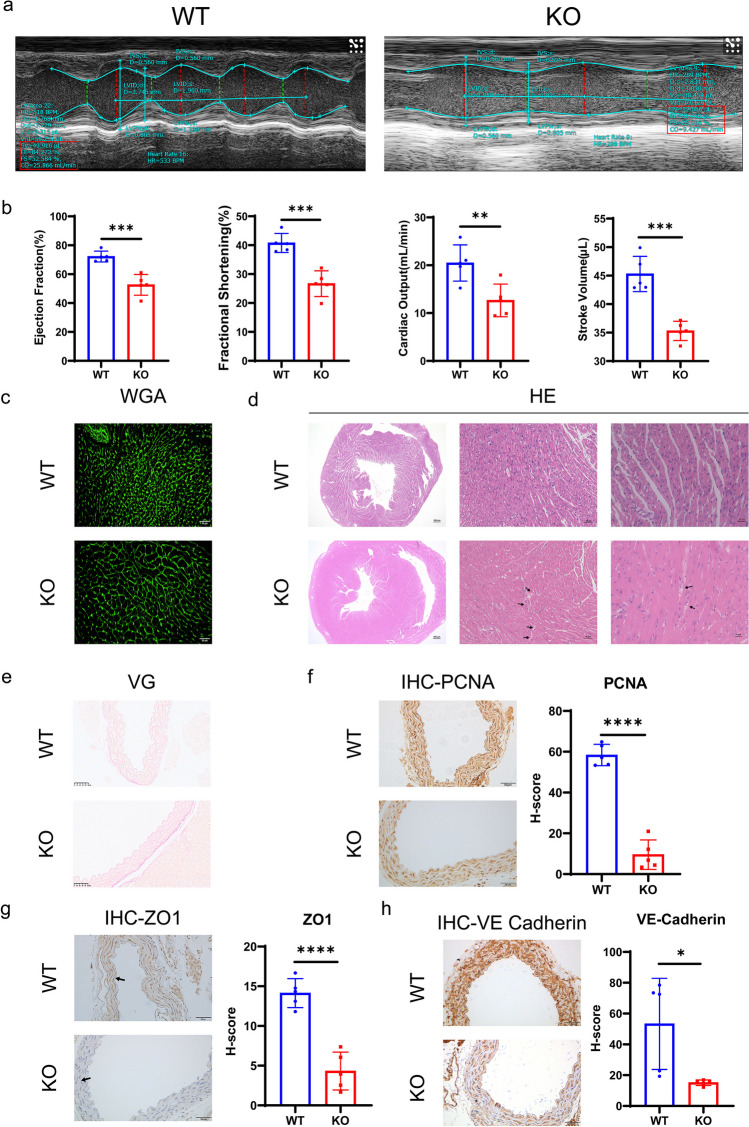
Cardiovascular complications in the allopurinol-rescued *Uox*^-/-^ mice at eight weeks after birth a. Representative images of M-mode echocardiography; b. Echocardiographic parameters of cardiac function; c. Representative images of WGA staining of the heart tissues; d. Representative images of HE staining of the heart tissues; e. Representative images of VG staining of the aorta; f. Aortic expression of proliferating cell nuclear antigen determined by immunohistochemistry; g. Aortic expression of Zo1 determined by immunohistochemistry; h. Aorta expression of Ve-cadherin determined by immunohistochemistry. Scale bar = 50μm; *n*=5 (four female, one male). Data were expressed as mean ±SEM; *: *P* < 0.05, **: *P* < 0.01, ***: *P* < 0.001, ****: *P* < 0.0001

Echocardiography showed markedly reduced ejection fraction (*P*=0.0097), fractional shortening (*P*=0.0084), cardiac output (*P*=0.0071), and stroke volume (*P*=0.0008) in *Uox*^-/-^ mice as compared to WT mice (Fig. 4a-b). Although, changes in echocardiographic dimensional parameters were not significant (Supplementary Fig. 1), histological examination revealed left ventricular wall thickening and decreased ventricular cavity in the *Uox*^-/-^ mice. Microscopic changes included hypertrophic cardiomyocytes with cytoplasmic vacuolation and myofibrillar fragmentation (Fig. 4c and d).

In addition, there were multiple pathological changes in the aorta of *Uox*^-/-^ mice. As compared to WT controls, there were more abundant aortic collagen fibers in the outer layer detected by VG staining (Fig. 4e). Moreover, the PCNA positive cells were less frequent (*P*<0.0001), indicating decreased proliferation of aortic endothelial cells (Fig. 4f); both ZO-1 (*P*<0.0001) and VE-cadherin (*P*=0.0270) expression were also significantly down-regulated in the aortic endothelial cells, suggesting that vascular endothelial barrier was compromised in the hyperuricemic mice (Fig. 4g and h).

### Liver Injury

**Fig. 5:**
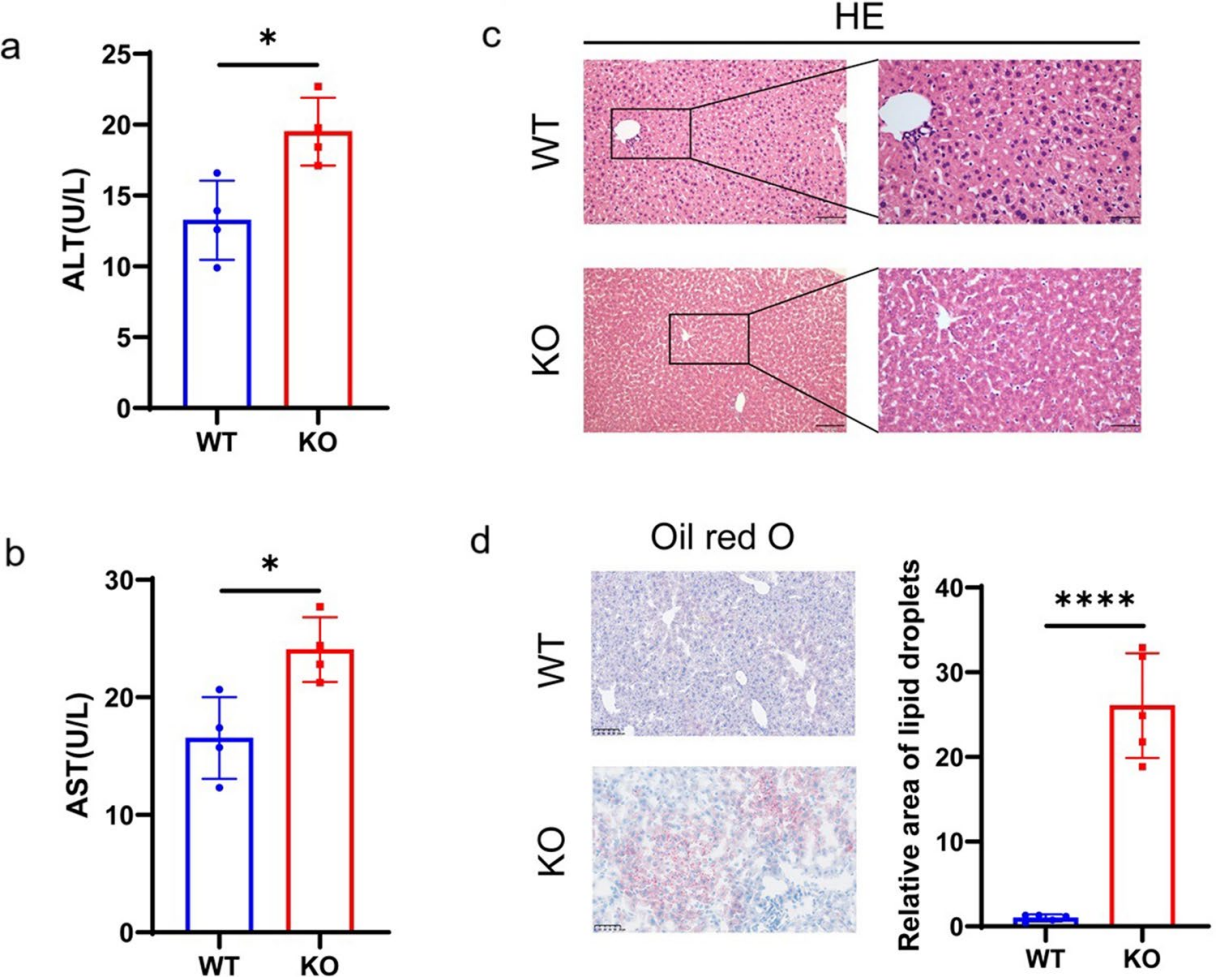
Liver injury in the allopurinol-rescued *Uox*^-/-^ mice at eight weeks after birth a. Serum concentration of alanine aminotransferase (ALT); b. Serum concentration of aspartate aminotransferase (AST); c. Representative images of HE staining of the liver tissues; d. Representative images of Oil red O staining of the liver tissues (*n*=5). Scale Bar=100μm; *n*=5 (four female, one male). Data were expressed as mean ±SEM. *: *P* < 0.05, ****: *P* < 0.0001

Hepatocyte injury in *Uox*^-/-^ mice was evidenced by the elevated levels of serum AST (*P*=0.0146) and ALT (*P*=0.0145) enzymes (Fig. 5a-b). Pathohistological changes included significantly widened sinusoidal space, enlarged hepatocytes with lightly stained cytoplasm and nuclei, as well as cellular ballooning (Fig. 5c). Oil-red O staining detected highly accumulated lipid droplets in the liver of *Uox*^-/-^ mice in comparison with their WT counterparts (*P*<0.0001, Fig. 5d).

## Discussion

Despite many efforts to model hyperuricemia in human, a suitable mouse model that have stable hyperuricemia and normal lifespan has been particularly lack [9]. The present study introduced a novel *Uox* deficient mice with spontaneous hyperuricemia generated by CRISPR/Cas9 system on a pure C57BL/6J background. To improve postnatal survival, we also applied a strategy that combined genetic manipulation with allopurinol intervention, which enabled the SUA concentration not lethally high but maintain valid that mimics “human like” hyperuricemia. Moreover, this allopurinol-rescued *Uox*^*-/-*^ mice presented various complications including kidney disease, metabolic syndrome, cardiovascular disease and NAFLD. The present mouse model could provide a candidate tool to study early-onset hyperuricemia and associated comorbidities.

Hyperuricemia is caused by hepatic overproduction and (or) renal and (or) intestinal underexcretion of SUA, which are regulated by genetic and environmental factors and their interactions [10]. Obviously, owing to the evolutionary inactivation of *Uox* in humans, genetic modification of rodent *Uox* is an essential and most effective way to replicate human hyperuricemia and urate biology, as compared to genetically engineered mice targeting other loci (for example, SLC2A9 and ABCG2), as well as environmentally induced models [9, 11–13].

The first *Uox* knockout model was generated on hybrid genetic background mouse (C57BL/6J*129Sv) by Neomycin-cassette insertion into exon 3 of the *Uox* that resulted in resulted in an elevated SUA concentration of 650 μmol/L, 12 times higher than that in the WT controls [6]. Lately, the second *Uox*^*-/-*^ mice was established on a C57BL/6J background by deleting 28 bp of exon 3 using TALEN technique, and the SUA levels were at 420 to 520 μmol/L, two to three times higher than in WT mice [7]. At present, ours represents the third *Uox* knockout mouse model so far, which has been generated on C57BL/6J background using CRISPR/Cas9 system for the first time. The exon 2 to exon 4 were targeted, which account for about 46% of the entire Uox protien coding gene. By deleting a bigger region (3296 bp) than the other models [6, 7], a frameshift mutation was expected with more significant phenotypic effect. In addition, deleting these three exons would result in less N-terminal amino acid residue. Beside, targeting other exons could affect the *Dnase2b,* since they coincide with the exons of *Dnase2b.* What’s more, off-target effect was highly unlikely given that their offspring presented the expected and stable biological features. Consequently, our *Uox*^*-/-*^ mice reached an extremely high SUA concentration of 1351.04±276.58 μmol/L, which was 5.5-fold higher than that in WT mice (248.19±100.59μmol/L), while allopurinol-treated *Uox*^*-/-*^ mice had moderately elevated SUA (612.55±146.98μmol/L), 2.5-time higher than that in WT mice. Due to lack of standard protocol for blood sampling and urate measurement, these reported SUA values varied widely without a clear definition of normal range in mice, and therefore were not directly comparable. Even though, the proportional increase in SUA suggested that present *Uox*^*-/-*^ mice yield a strong phenotypic effect on SUA level.

Without a gradual adaption to the evolutionary changes in urate mediated biological system [9], acute disruption in the Uox gene may largely explain the substantial embryonic and postnatal mortality in mice. The percentages of the *Uox*^*-/-*^ mice born from heterozygous mating were reported at 7.1% [6] and 15.9% [7], respectively, both far below the expected Mendelian frequency. By contrast, the birth rate in present *Uox*^*-/-*^ mice is 21.4%, approximated to the expected rate of 25%, suggesting the advantage of CRISPR/Cas9 technique in reducing embryonic lethality. However, neonatal mortality at four weeks after birth was extremely higher (92%) in the present model than the previous reports (40%~65%) [6, 7]. The same genetic approach targeting at exon 2-4 of *Uox* on Sprague Dawley rats resulted in a 95% survival up to one year, but only mild hyperuricemia, suggesting different responses to *Uox* inactivation between animal species [8]. Clearly, the extremely high SUA is the fundamental cause of premature death in the present *Uox*^*-/-*^mice. With a 55% reduction in SUA by allopurinol intervention, they all could survive to eight weeks to be sexually matured.

The kidney is one of the important organs of urate metabolism, and studies have shown that patients with hyperuricemia are at increased risk of renal disease [3, 13]. As previous hyperuricemic mouse models [6, 7, 14, 15], our experimental results demonstrated severe renal insufficiency evidenced by remarkably increased levels of blood urea and serum creatinine, significant glomerular and tubular deformations accompanied by corticomedullar inflammation, interstitial fibrosis and uric acid crystal deposition. Given the fact that SUA concentration was even doubled in the *Uox*^*-/-*^ mouse prototype, we assume that severe urate nephropathy and renal failure in the first four weeks of life may account for their poor survival.

Epidemiological and experimental studies have shown that hyperuricemia is associated with hypertension, coronary atherosclerosis and heart failure [16–23]. However, the causal relationship between UA and cardiovascular disease remains controversial. Our experimental mouse model of hyperuricemia manifested hypertension along with distinct ventricular remodeling, reduced cardiac output and significant aortic endothelial dysfunction, which are consistent with the literature [21–23]. Owing to the renal failure, the independent role of hyperuricemia in the development of hypertension remains uncertain. Likewise, both kidney disease and hypertension may contribute to cardiac dysfunction and atherosclerosis, reiterating the issue of inconclusive causal relationship between hyperuricemia and cardiovascular disease. Notwithstanding, such a complex links enables our *Uox*^*-/-*^ mice as an appropriate model to study the progression trajectories of these comorbidities from birth to adulthood. Another appealing use is to detect whether new urate lowering drugs in addition to allopurinol could further ameliorate early-onset hypertension and cardiovascular complications as compared to allopurinol alone.

In addition to hypertension, our experimental mouse model of hyperuricemia manifested distinct hyperglycemia and dyslipidemia, all of which are typical to metabolic syndrome [18]. Onset sequence study showed that hyperuricemia is an earlier-onset metabolic disorder in relation to hypertension, hypertriglyceridemia, and diabetes mellitus, indicating a potential upstream role of hyperuricemia in the development of metabolic syndrome [1]. It was reported that high SUA could directly cause pancreatic β-cell apoptosis and dysfunction [24, 25]**.** The previous results from *Uox*^*-/-*^ mice also supported hyperuricemia was probably a causal factor of islet dysfunction and therefore diabetes [7]. Whether hyperuricemia also induces insulin resistance in *Uox*^*-/-*^ mice warrants further investigation.

Regarding lipid metabolism, our *Uox*^-/-^ mice displayed so-called atherogenic lipid triad: markedly increased levels of TC, TG and LDL-C, as well as decreased levels of HDL-C, although not reaching a statistical significance except for TC. The underlying mechanism is unclear. One possible explanation is that knocking out of *Uox* may cause hepatocellular injury, as evidenced in our *Uox*^-/-^ mice, thereby disturbing hepatic lipid metabolism [25]. On the other hand, excessive influxes of lipids could promote fat accumulation and NAFLD, which has been widely recognized as a cardiometabolic disorder [26]. NAFLD is speculated to cause hyperuricemia by inducing the expression of hepatic xanthine oxidase without changing *Uox* expression [27, 28]. Interestingly, an opposite trend was observed in our *Uox*^*-/-*^ mice, in which NAFLD was evidenced by hepatic steatosis and hepatocellular ballooning, suggesting hyperuricemia as a potential driving cause of NAFLD. This was supported by previous experimental studies showing UA can induce hepatic steatosis in HepG 2 cells [29].

The present model has important shortfalls. To begin, we lack direct autopsy evidence for major cause of premature death in prototype *Uox*^*-/-*^ mice. Moreover, only 50% of allopurinol-rescued *Uox*^*-/-*^ mice survived to 12 weeks, limiting the use of current model in the long-term studies. However, the spontaneous high levels of SUA and multiple organ injuries actually replicate the clinical patients of early-onset hyperuricemia with severe complications. Another major limitation is that we failed to exam phenotypic sex disparities due to limited sample size [9]. Confounding factors due to sex bias should be considered when interpreting these study results. Most importantly, the use of allopurinol should be titrated in a dose and time dependent manner and dynamic observations of biological properties of our modified *Uox*^*-/-*^ mice is necessary in the future.

## Conclusions

In summary, this is a novel mouse model of hyperuricemia established in two steps: knocking out of *Uox* by CRISPR/Cas9 system (prototypic *Uox*^*-/-*^ mice) and allopurinol administration to improve their survivals up to 16 weeks (modified *Uox*^*-/-*^ mice). Given the significantly elevated SUA along with urate nephropathy, cardiovascular disease and metabolic syndrome, the present modified *Uox*^*-/-*^ mice could be suitable candidate for short and mid-term studies on early-onset hyperuricemia with severe complications. The model is useful to improve our understanding the hyperuricemia mediated cardiovascular and cardiometabolic disorders, as well as to develop more efficient novel therapies.

## Supplementary Information

ESM 1 (DOCX 140 kb)

## Data Availability

The datasets generated during and/or analysed during the current study are available from the corresponding author on reasonable request.

## Abbreviations

ALT: Alanine aminotransferase
AST: Aspartate aminotransferase
BP: Blood pressure
cDNA: Complementary DNA
HDL-C: High density lipoprotein cholesterol
HE: Hematoxylin-eosin
IQR: Interquartile ranges
LDL-C: Low-density lipoprotein cholesterol
NAFLD: Nonalcoholic fatty liver disease
PCNA: Proliferating cell nuclear antigen
PCR: Polymerase chain reaction
SUA: Serum uric acid
SEM: Standard error of mean
TC: Total cholesterol
TG: Triglyceride
UA: Uric acid (UA)
UOX: Urate oxidase
VG: Van Gieson
WGA: Wheat Germ Agglutinin
WT: Wild type (WT)
