# Supplementary material for: CRISPR/Cas9 Mediated Deletion of the Uox Gene Generates a Mouse Model of Hyperuricemia with Multiple Complications
Source: J Cardiovasc Transl Res. 2024 Jun 10;17(6):1455–65. doi: 10.1007/s12265-024-10526-6 (PMC11635051; doi:10.1007/s12265-024-10526-6)

**Supplementary Materials**

**The protocol for genotyping of mice：**

The primer sequences for wild type genotype:

5’-GCAATGAACATCTGTGAGCACTTC-3’ (forward)

5’-ACCTGCCGACTCTTGAAGAATAG-3’ (reverse)

The primer sequences for *Uox*^-/-^ genotype:

5’-GGCTCTGCATTGCTGAGAGTATTC-3’ (forward)

5’-AATGGGACACCAGCTTTAGGAG-3’ (reverse)

PCR reaction protocol: initial denaturation at 94°C for 5min, 35 cycles at 94°C for 30s, 60°C for 30s, 72°C for 30s, and a final extension at 68°C for 5min.

**Supplementary Fig. 1 Dimensional parameters determined by M-mode echocardiography at in the allopurinol-rescued Uox-/- mice at eight weeks after birth**

Dimension of left ventricular posterior wall (LVPW); B. Left ventricular internal dimension (LVID); C. Dimension of interventricular septum (IVS) ; D: Left ventricular volume;E. Left ventricular mass;(F). Heart rate. Note: d=diastolic; s=systolic; WT:wild type; KO=*Uox* knockout mice;n=5 (four female, one male)**;** data were expressed as mean ±SEM; ** represents for P < 0.01.


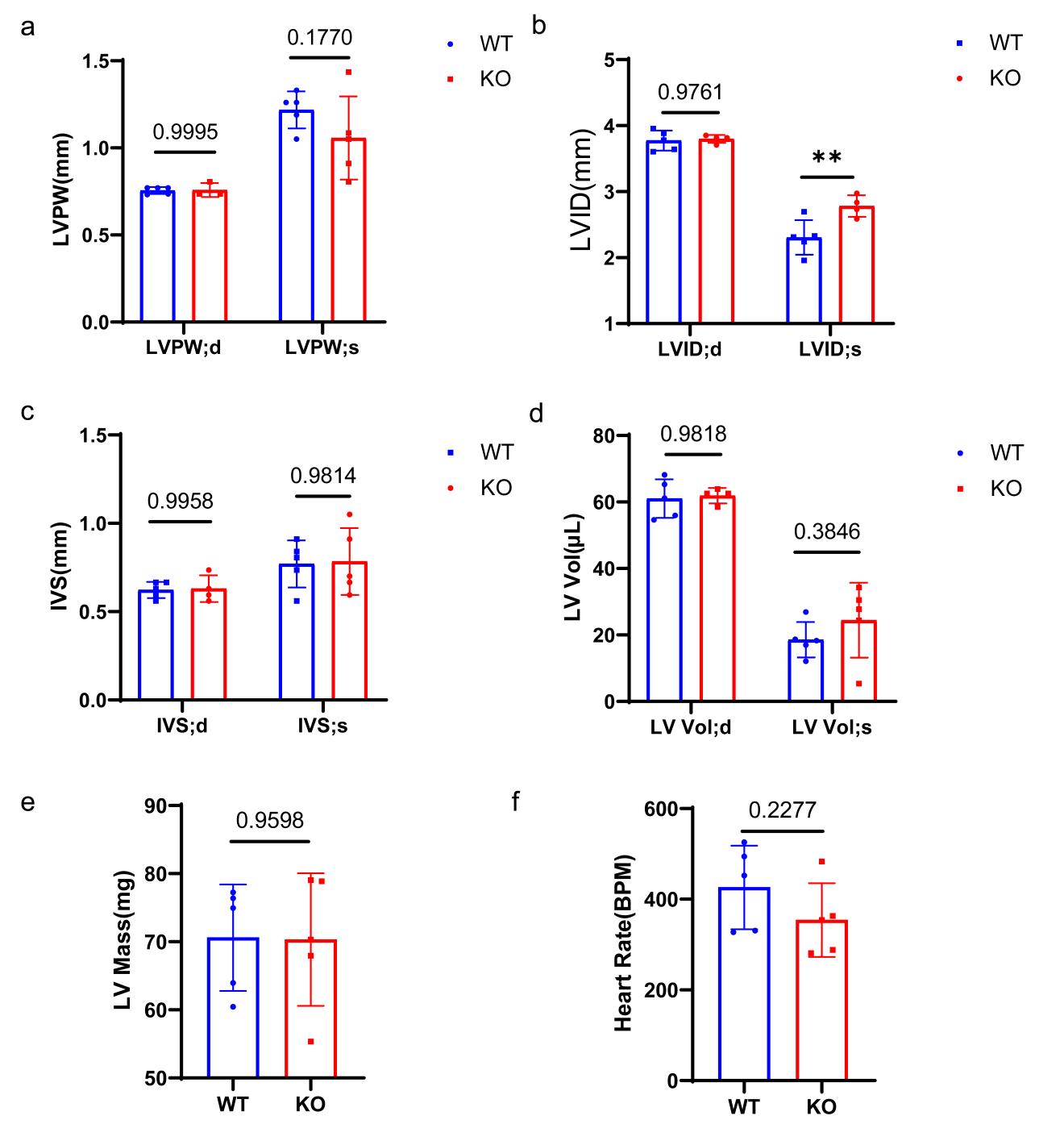

Supplement: Supplementary file 1 — (DOCX 140 kb) [file 12265_2024_10526_MOESM1_ESM.docx]
